# Induced normothermia ameliorates the procoagulant host response in human endotoxaemia

**DOI:** 10.1016/j.bja.2021.02.033

**Published:** 2021-04-23

**Authors:** Matthew B.A. Harmon, Nanon F.L. Heijnen, Sanne de Bruin, Niek H. Sperna Weiland, Joost C.M. Meijers, Anita M. de Boer, Marcus J. Schultz, Janneke Horn, Nicole P. Juffermans

**Affiliations:** 1 Laboratory of Experimental Intensive Care and Anaesthesiology, Amsterdam University Medical Centers, University of Amsterdam, Amsterdam, the Netherlands; 2 Department of Intensive Care Medicine, Amsterdam University Medical Centers, University of Amsterdam, Amsterdam, the Netherlands; 3 Department of Anaesthesiology, Amsterdam University Medical Centers, University of Amsterdam, Amsterdam, the Netherlands; 4 Department of Experimental Vascular Medicine, Amsterdam University Medical Centers, University of Amsterdam, Amsterdam, the Netherlands; 5 Department of Molecular and Cellular Haemostasis, Sanquin, Amsterdam, the Netherlands; 6 Mahidol Oxford Research Unit, Mahidol University, Bangkok, Thailand; 7 Nuffield Department of Medicine, University of Oxford, Oxford, UK; 8 Department of Intensive Care Medicine, OLVG Hospital, Amsterdam, the Netherlands

**Keywords:** coagulation, cooling, endotoxaemia, fever, induced normothermia, inflammation, sepsis

## Abstract

**Background:**

Dysregulation of coagulation occurs commonly in sepsis, ranging from mild coagulopathy with decreased platelets to disseminated intravascular coagulation (DIC). We investigated the effect of induced normothermia on coagulation during lipopolysaccharide (LPS)-induced endotoxaemia in healthy volunteers.

**Methods:**

Twelve volunteers received an infusion of bacterial lipopolysaccharide (*Escherichia coli*; 2 ng kg^−1^) and were assigned to either induced normothermia or control. Induced normothermia to maintain core temperature at 37°C consisted of external surface cooling, cold i.v. fluids, and medication to reduce shivering (buspirone, clonidine, and magnesium sulphate). The primary outcome was the DIC score (International Society on Thrombosis and Haemostasis guideline). Prothrombin time (PT), activated partial thromboplastin time (aPTT), D-dimer, plasma von Willebrand factor (vWf), and rotational thromboelastometry (ROTEM) were measured before and 1, 3, 6, and 8 h after LPS infusion. Differences between groups were tested with a mixed effects model.

**Results:**

In control subjects, lipopolysaccharide caused a fever, transiently decreased platelet levels and lowered activated partial thromboplastin time, while prolonging prothrombin time and increasing D-Dimer and vWf levels. Normothermia prevented the DIC-score exceeding 4, which occurred in 50% of control subjects. Normothermia also reduced the fall in platelet count by 67x10^9^ L^−1^([95%CI:27-107]; p=0.002), aPTT (mean difference:3s [95%CI:1-5]; p=0.005) and lowered vWf levels by 89% ([95%CI:6-172]; p=0.03), compared to the fever group. ROTEM measurements were unaffected by lipopolysaccharide.

**Conclusion:**

In human endotoxaemia, induced normothermia decreases markers of endothelial activation and DIC. Maintaining normothermia may reduce coagulopathy in hyperinflammatory states.

**Editor's key points**

• Normothermia may limit the activation of coagulation in sepsis, although proof-of-concept studies in humans are lacking.
• Twelve volunteers received a bacterial lipopolysaccharide infusion with or without induced normothermia.
• Induced normothermia prevented fever, reduced platelet count, and reversed other measures indicative of activation of coagulation after lipopolysaccharide infusion, including subclinical DIC.
• Maintaining normothermia may reduce coagulopathy in hyperinflammatory states such as sepsis.

The dysregulated host immune response in sepsis results in activation of coagulation, microthrombi formation, and consumption of platelets and coagulation factors. These pathological features are reflected by low platelet count, prolonged prothrombin time (PT), increased D-dimer concentrations and overt disseminated intravascular coagulation (DIC) in severe sepsis, associated with multiple organ failure.^1^ Sepsis often presents with fever, defined as a core body temperature of >38.3°C.^2^ Fever is commonly thought to be a functional response to infection as it improves pathogen clearance and immune cell mobility.^3^ However, fever may also have harmful effects. The increased metabolic cost of fever may compromise cellular oxygenation, promoting multiple organ injury.^3^ Fever may also contribute to an exaggerated proinflammatory response, with increased cytokine production and vasodilatory shock. In patients with septic shock presenting with fever, induced normothermia reduced vasopressor requirements,^4^ although not all studies have reported beneficial effects.^5^

While profound hypothermia decreases platelet function and impairs both the synthesis and kinetics of clotting factors,^6^ induced normothermia may both limit inflammation^5^ and reduce activation of coagulation. However, proof-of-concept data are lacking on how cooling affects the procoagulant host response. Experimental human endotoxaemia provides a controlled setting to study the effects of fever control on the host immune response. The model of endotoxaemia induced by lipopolysaccharide (LPS) is characterised by a procoagulant response, with increased concentrations of von Willebrand factor (vWf) and tissue factor, together with a (transient) decrease in platelet count and prolonged PT, thereby closely resembling sepsis-associated coagulopathy.^7^, ^8^, ^9^

In this study, we investigated the effect of induced normothermia on coagulation during LPS-induced endotoxaemia in healthy volunteers. We hypothesised that cooling to normothermia would limit the endothelial-mediated procoagulant host response.

## Methods

### Study design

This study was reviewed and approved by the Amsterdam University Medical Centre Medical Ethical Committee (NL53460.018.15) and performed according to the Declaration of Helsinki, including Good Clinical Practice. The External Surface Cooling In huMan endOtoxemia (ESCIMO) study was a human volunteer open-label, non-RCT conducted at the Amsterdam University Medical Centre.

#### Inclusion criteria

Healthy male volunteers >18 yr with unremarkable medical history and physical examination were eligible.

### Procedures

Every subject received an indwelling arterial catheter, placed in the radial artery of the subjects' non-dominant arm, for measurement of BP and blood sampling. A rectal temperature probe was inserted to measure core temperature. Other vital parameters were monitored with ECG and pulse oximetry. Ambient temperature in the room was set at 21°C. All subjects received *Escherichia coli* LPS (National Institutes of Health Clinical Centre, Bethesda, MD, USA) 2 ng kg^−1^.

### Sample collection and analysis and outcomes

Citrated blood samples (BD™ Vacutainer™ Citrate Tubes, Becton, Dickinson and Company, UK) were obtained from the arterial catheter just before and at 1, 3, 6, and 8 h after LPS administration. Blood was centrifuged at 1500 g and the supernatant was stored at –80°C for later analysis of vWf antigen analysis by enzyme-linked immunosorbent assay using a homemade assay with antibodies from DAKO (Glostrup, Denmark), with a standard curve using standard human plasma from Siemens calibrated to WHO International Standard 07 316 and a coefficient of variation of 6%.

### Rotational thromboelastometry

Citrated whole blood was analysed with the rotational thromboelastometry (ROTEM) delta device at 37°C. The variables measured were: clotting time (CT), clot amplitude (CA) after 5 (CA5), 10, 15, 20, 25, and 30 min, α-angle (alpha), maximum clot firmness (MCF), clot lysis at 30, 45, and 60 min, and the maximum lysis in percentage (ML). If an error appeared during the test or it seemed that a subtest (such as extrinsically activated test [EXTEM], intrinsically activated test [INTEM], or fibrin-based extrinsically activated test [FIBTEM]) did not run properly, that particular test was repeated immediately with blood retrieved from the same sample in order to provide a reliable result. The G value was assessed using the formula (5000×MCF) (100–MCF) and expressed as dynes cm^2^.^10^ The DIC score was calculated according to the International Society on Thrombosis and Haemostasis (ISTH) guideline.^11^

### Protocol

The first six participants received LPS alone (control [fever] group). The following six participants were included in the normothermia group who received LPS and who were subsequently cooled to normothermia. In the normothermia group, cooling was initiated 1 h after commencing LPS infusion for 7 h using an external surface cooling device (Arctic Sun© temperature management system, Becton, Dickinson and Company, UK), set at a target temperature of 36.0–37.0°C. To counteract shivering and thermal discomfort during cooling, buspirone 30 mg was given orally at the initiation of induced normothermia. In addition, the subjects received clonidine (75 μg bolus followed by a continuous infusion of 1–2 μg kg^−1^ h^−1^) and magnesium sulphate (4 g bolus followed by a maximum continuous infusion of 2 g h^−1^ for 150 min).^12^^,^^13^ To prevent nausea, ondansetron 4 mg was given i.v.

#### Primary outcome

The primary outcome was DIC score.

#### Secondary outcomes

We also analysed prothrombin time (PT)/ activated partial thromboplastin time (aPTT), D-dimer, fibrinogen, platelet count, vWf antigen concentrations, and ROTEM parameters.

### Sample size estimation

Statistical analyses were performed using Version 1.2.1335 (R Studio Team (2020). RStudio: Integrated Development for R. RStudio, PBC, Boston, MA). As no previous study has examined the effect of fever control on LPS-induced endotoxaemia in humans, a formal power calculation was not possible. As a proxy of inflammation-driven coagulopathy, interleukin-6 (IL-6) was used. In a previous study in rats^14^, fever control resulted in a decrease in plasma IL-6 concentrations compared with controls (35 [5] ng ml^−1^ *vs* 130 [30] ng ml^−1^). Thereby, *n*=3 volunteers per group assuming an alpha of 0.05, would have >80% power to detect decreases in IL-6. As the primary outcome was different and our study is in humans with larger variation, we included *n*=6 per group.

### Statistical analysis

Depending on normality of the data, baseline differences between groups were calculated with either the Student's *t*-test or the Wilcoxon ranked sums test. To compare changes over time within groups, a paired Student's *t*-test or the Wilcoxon ranked sums test was performed between T=0 and the maximum or minimum value during the study period. Linear mixed models were used to analyse differences in continuous variables between groups over time, using time point and group as fixed effects and subject ID as random effect. Nested models with and without group as a variable were compared to determine differences between groups. If data were non-parametric, data were transformed before statistical testing. Normally distributed data were presented as mean (standard deviation). Non-parametric data were presented as median (25–75th percentile). Results from the linear mixed models were presented as β-coefficient (β) and 95% confidence interval (95% CI). A *P*-value below 0.05 was considered statistically significant.

## Results

### Study participants

Twelve healthy male volunteers aged 18–35 yr participated.

### Physiological response to LPS infusion

Mean peak temperature increased to 38.7°C (0.3) 3 h after the start of the LPS infusion, whereas normothermia maintained normal body temperature (mean peak temperature at T=3 h 37.2°C [0.3]; *P*<0.0001). LPS increased HR from 59 (6) beats min^−1^ at baseline to 93 (11) 4 h after the start of the infusion. Over 8 h of LPS infusion, peripheral leukocyte count increased from 5.5 (1.5)×10^9^ L cells at baseline to 13.7 (3.2)×10^9^ L cells, accompanied by an increase in C-reactive protein from 0.4 (0.3–0.9) mg ml^−1^ at baseline to 7.7 (6.9–10.0) mg ml^−1^.

### Primary outcome

**Fig 1:**
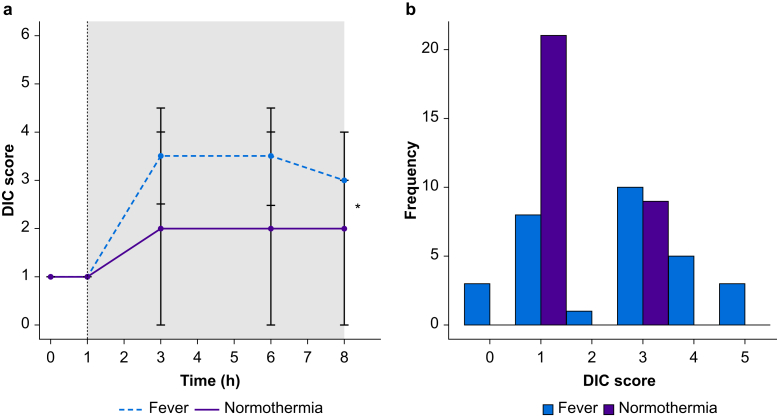
DIC scores in volunteers with endotoxaemia and fever and volunteers with endotoxaemia treated with induced normothermia. a shows DIC scores over time. Lipopolysaccharide was given to all volunteers at T=0. Cooling to normothermia (37°C) was initiated at T=1 in the normothermia group. The grey box in Fig 1a represents the period of induced normothermia. The dashed line represents the fever group and the solid line represents the normothermia group. Data are presented as medians with bars representing the inter-quartile ranges. b shows the frequency of DIC scores in both groups over all time points. DIC, disseminated intravascular coagulation. ∗*P*<0.05, N.S., not significant.

Calculated ISTH DIC scores were lower in subjects receiving the normothermia intervention, compared with the control group (Fig 1a). Half of the subjects in the fever group reached a DIC score above 4 at some time point during the study period, whereas none of the subjects in the normothermia group reached a DIC score above 4 (Fig 1b).

### Secondary outcomes

#### Prothrombin/activated partial thromboplastin time

**Fig 2:**
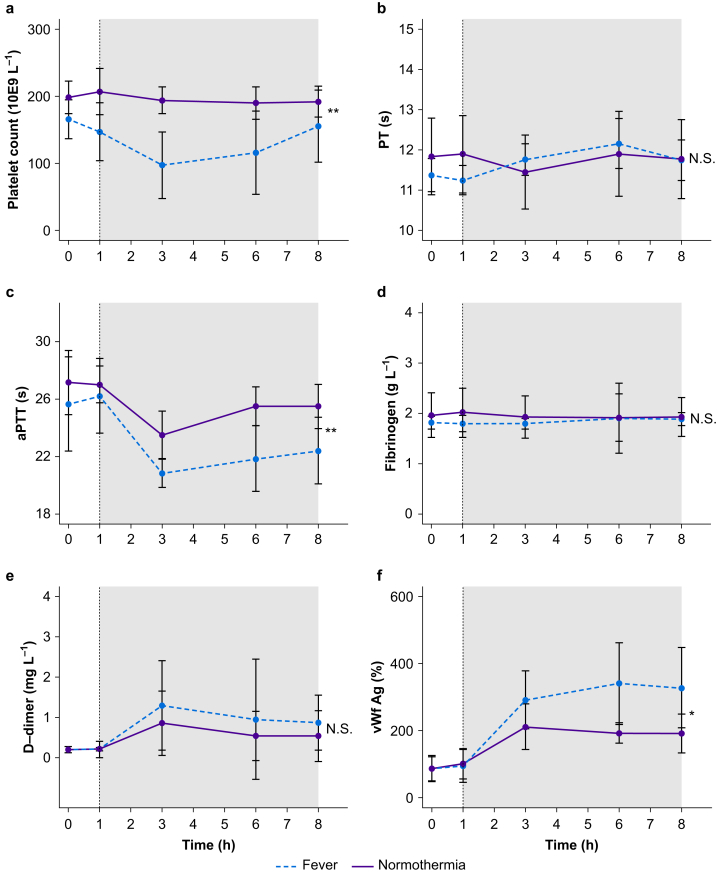
Conventional coagulation tests over time in volunteers with endotoxaemia and fever and volunteers with endotoxaemia treated with induced normothermia. Lipopolysaccharide was given to all volunteers at T=0. Cooling to normothermia (37°C) was initiated at T=1 in the normothermia group. The grey box represents the period of induced normothermia. The dashed line represents the fever group and the solid line represents the normothermia group. a–e: data are presented as means with standard deviation. f: data are presented as medians with inter-quartile range. aPTT, activated partial thromboplastin time; PT, prothrombin time; vWf Ag, von Willebrand factor antigen. ∗*P*<0.05, ∗∗*P*<0.01, N.S., not significant.

LPS prolonged PT in the control group, but remained unaltered in subjects allocated to normothermia (Fig 2b). LPS decreased aPTT from 25.7 (3.3) s at baseline to 20.8 (1.0) s at T=3 h, which was prevented in the normothermia group (Fig 2c). LPS did not alter fibrinogen concentrations (Fig 2d).

#### Platelet count/vWf antigen concentrations

LPS resulted in thrombocytopenia, but induced normothermia resulted in higher platelet counts (Fig 2a). LPS increased vWf antigen concentrations, which was prevented by induced normothermia (Fig 2f).

#### D-dimer

LPS infusion resulted in an increase in D-dimer 3 h after the LPS infusion, which persisted until the end of the study period. D-dimer values were similar between control and induced normothermia (Fig 2e; Supplementary Table S1).

#### ROTEM

**Fig 3:**
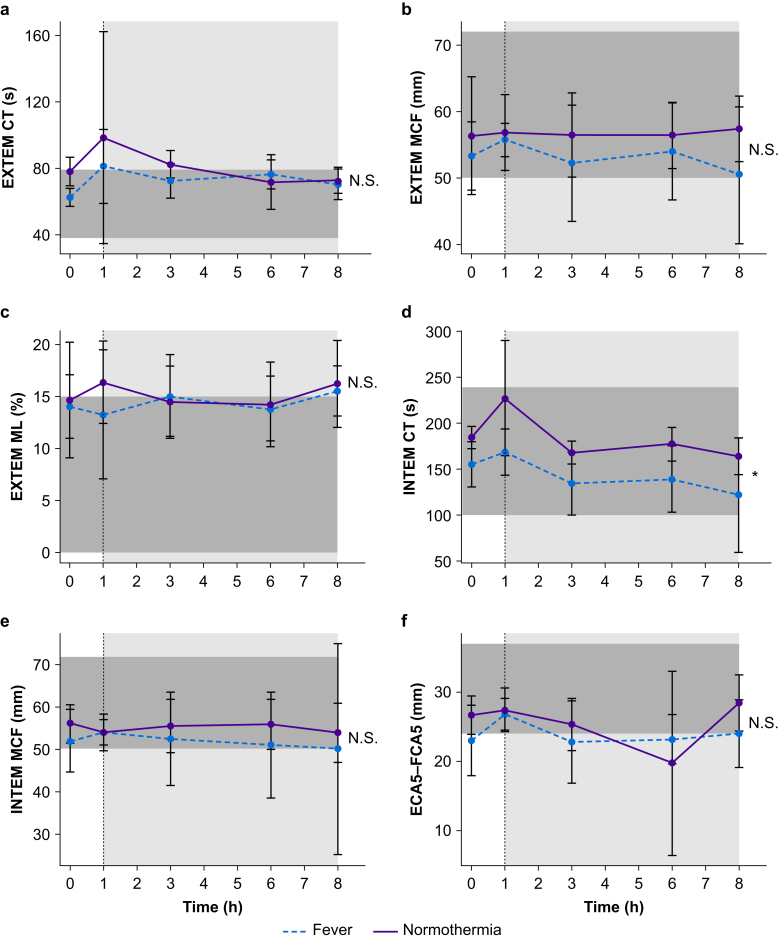
Clot formation and lysis parameters of rotational thromboelastometry (ROTEM) in volunteers with endotoxaemia and fever and volunteers with endotoxaemia treated with induced normothermia. Lipopolysaccharide was given to all volunteers at T=0. Cooling to normothermia (37°C) was initiated at T=1 in the normothermia group. The light grey box represents the period of normothermia. The dark grey box represents the reference range for specific ROTEM tests. The dashed line represents the fever group and the solid line represents the normothermia group. Data are presented as means with standard deviation. CT, clotting time; DIC, disseminated intravascular coagulation; EXTEM, extrinsically activated test; ECA5-FCA5, EXTEM clot amplitude after 5 min–fibrin-based extrinsically activated test (FIBTEM) clot amplitude after 5 min. INTEM, intrinsically activated test, MCF, maximum clot firmness; ML, maximum lysis. ∗*P*<0.01, N.S., not significant.

There was a large variation in ROTEM values, and these largely remained within normal reference ranges. (Fig 3; Supplementary Table S2). INTEM CT was higher after induced normothermia compared with the control group (Fig 3d). Differences in INTEM CT concentrations were observable 1 h after starting LPS infusion, before cooling was initiated. Despite differences in platelet concentrations between control and induced normothermia groups, EXTEM CA5–FIBTEM CA5 (a measure of platelet function) were similar (Fig 3f).

## Discussion

The main finding of this study is that induced normothermia reversed LPS-induced thrombocytopenia and DIC scores compared with subjects who developed fever. Elevated vWf antigen concentrations were also decreased. Normothermia appears to reverse derangements in coagulation, as seen during sepsis-induced coagulopathy.

LPS-induced endotoxaemia in human volunteers resulted in derangements of the coagulation system resembling those noted in sepsis-induced consumption coagulopathy and DIC. The coagulation abnormalities induced by LPS are similar with previous studies on sepsis-induced coagulopathy, including activation of the endothelium and increased secretion of vWf, resulting in activation of platelets.^15^ These platelets have a high capacity to aggregate, causing clumping of platelets with an ensuing decrease in circulating platelets.^16^^,^^17^ LPS also prolonged PT, presumably as a result of activation and consumption of coagulation factors in the formation of microthrombi. Together, LPS induces an increase in DIC scores.^18^ The decrease in aPTT after LPS is most likely caused by coagulation factor VIII as LPS is known to increase factor VIII.^19^

Induced normothermia prevented several LPS-induced derangements in coagulation. Induced normothermia maintained platelet concentrations. Overall, prolongation of PT and increase in D-dimer levels were less outspoken in the normothermia group compared to the fever group. This resulted in decreased DIC scores after induced normothermia, and in a reduction of the number of volunteers with severe DIC scores. Of note, fibrinogen concentrations were unaffected by cooling. In LPS-induced endotoxaemia, fibrinogen concentrations increase after 24 h, beyond the time frame of this study.^19^

Induced normothermia may reduce DIC through an effect on the endothelium, as we found that cooling to normothermia reduced concentrations of vWf which is a marker of endothelial activation.^19^, ^20^, ^21^ Our study cannot dissect cause from effect. Induced normothermia may inhibit the consumption of platelets and coagulation factors and subsequent microthrombi formation, which may have prevented endothelial damage.^19^ However, this may not be in line with the finding that LPS-induced thrombocytopenia is transient. A more likely explanation is that induced normothermia reduced endothelial cell activation with less activation of the ensuing coagulation cascade. The effect on the aPTT may also be explained by less endothelial cell activation with less vWf release. As vWf is the carrier of factor VIII in plasma, decrease in vWf in the normothermia group compared with controls may in part explain normalisation of aPTT concentrations in the normothermia group.

Consistent with the decrease in aPTT, INTEM CT in controls was prolonged compared with induced normothermia. Notably, the differences in INTEM CT between control and induced normothermia were already apparent at T=1, so the differences between groups may not have been a result of normothermia. Other LPS-induced coagulation derangements were not detected by ROTEM results. As ROTEM values are influenced by platelet function and counts,^22^ and ROTEM detects DIC, this finding was unexpected.^22^, ^23^, ^24^ However, the impact of LPS on ROTEM values appears to be subtle, which may be attributable to the small number of subjects studied.

There are additional limitations to our study. To reduce shivering, medication including magnesium sulphate was used. Thereby, we cannot dissect whether the external cooling or the medications contributed to differences in coagulation. High magnesium concentrations inhibit blood coagulation and thrombus formation^25^ and potentially limit inflammation in experimental studies,^26^, ^27^, ^28^, ^29^ possibly augmented by the sympatholytic effects of magnesium by blocking norepinephrine release.^30^ Magnesium influences systemic inflammation, as shown by magnesium-deficient rats faced with an endotoxin challenge resulting in increased production of inflammatory cytokines.^29^ Magnesium also suppresses inflammatory markers in cells treated with LPS, possibly through antagonising calcium and L-type calcium channels.^26^ These effects may be augmented by the sympatholytic effects of magnesium, which also blocks norepinephrine release by blocking N-type calcium channels.^30^ An α2-adrenoreceptor agonist, such as clonidine, does not induce platelet aggregation^31^^,^^32^ or affect coagulation,^33^ but may also have anti-inflammatory effects.^31^^,^^32^^,^^34^ We attempted to include a third control group receiving only medication but without induced normothermia. Because of hypotension, we prematurely stopped inclusion in this group. However, as external cooling requires some form of sedation or analgesia to combat shivering, this study reflects pragmatic clinical practice. Lastly, for pragmatic reasons, we did not randomise subjects in this study.

In order to unravel the underlying mechanisms of induced normothermia on hyperinflammatory states, future studies may focus on omic analyses to determine specific pathways that may be orchestrating these effects on coagulation. Such analyses will not only provide mechanistic insights, but may also be used in the future to apply different temperature management strategies to patient-specific endotypes.

In summary, induced normothermia reverses LPS-induced derangements of the coagulation system, including markers of endothelial activation and DIC, suggesting a decrease in endothelial-driven coagulation. Induced normothermia should be further studied as a potential treatment in hyperinflammatory states such as sepsis.

## Authors' contributions

Designed the study: MH, SdB, MS, JH, NJ.

Performed the analyses: MH, NH, AdB

Collected the data: MH, NH, SdB, NSW, AdB, NJ

Drafted the manuscript: MH, NH, JM, NJ

Critically reviewed the manuscript: all authors

Read and approved the final version of this manuscript: all authors

## Declarations of interest

The authors declare that they have no conflicts of interest.
