# Supplementary material for: Induced normothermia ameliorates the procoagulant host response in human endotoxaemia
Source: Br J Anaesth. 2021 Apr 23;126(6):1111–8. doi: 10.1016/j.bja.2021.02.033 (PMC8258978; doi:10.1016/j.bja.2021.02.033)
Supplement: Multimedia component 1 [file mmc1.docx]

SUPPLEMENTAL MATERIAL

**Induced normothermia ameliorates the procoagulant host response in human endotoxemia.**

Matthew B.A. Harmon, MD, Nanon F.L. Heijnen, MD, Sanne de Bruin, MD, Niek H. Sperna Weiland, MD, Joost C.M. Meijers, PhD, Anita M. de Boer, Marcus J. Schultz, MD, PhD, Janneke Horn, MD, PhD, Nicole P. Juffermans MD, PhD.

**Supplemental Table 1:** Overview of results from the mixed effects models of conventional coagulation tests and derived coagulation abnormality scores comparing volunteers with endotoxemia and fever and volunteers with endotoxemia treated with induced normothermia (37°C).

| **Variable** | **Beta-coefficient** | **95% Confidence interval** | **P-value** |
| --- | --- | --- | --- |
| **Platelet count (10^9^/L cells)** | 67.1 | 27.1 - 107.2 | 0.002 |
| **Fibrinogen (g/L)** | 0.1 | -0.4 - 0.5 | 0.78 |
| **D-dimer (mg/L)** | -0.3 | -0.7 - 0.0 | 0.06* |
| **vWf (%)** | -89.2 | -171.7 - -6.6 | 0.03 |
| **aPTT (sec)** | 2.7 | 0.9 - 4.5 | 0.005 |
| **PT (sec)** | 0.0 | -0.8 - 0.8 | 0.99 |
| **G-value (dynes/cm2)** | 1.1 | -0.6 - 2.7 | 0.15 |
| **DIC-score** | -0.3 | -0.6 - 0.0 | 0.04* |

* Log transformed data.

Abbreviations: aPTT= activated partial thromboplastin time, DIC= disseminated intravascular coagulation, vWf= von Willebrand factor

**Supplemental table 2:** Overview of results from the mixed effects model analyses of the rotational thromboelastometry (ROTEM) tests comparing volunteers with endotoxemia and fever and volunteers with endotoxemia treated with induced normothermia (37°C).

|  | **EXTEM** |  |  | **INTEM** |  |  | **FIBTEM** |  |  |
| --- | --- | --- | --- | --- | --- | --- | --- | --- | --- |
| **Subtest** | **β** | **95% CI** | **P-value** | **β** | **95% CI** | **P-value** | **β** | **95% CI** | **P-value** |
| **Coagulation time (sec)** | 6.4 | -13.0 - 25.6 | 0.47 | 43.9 | 13.0 – 74.8 | 0.007 | 22.5 | -8.3 – 53.0 | 0.15 |
| **Maximum clot firmness (mm)** | 4.8 | -2.0 - 11.6 | 0.13 | 9.7 | -1.1 - 20.4 | 0.06 | 4.74 | -2.0 - 11.4 | 0.13 |
| **Amplitude 5 minutes (mm)** | 5.1 | -1.5 - 11.7 | 0.10 | 7.14 | -2.2 - 16.5 | 0.11 | 3.07 | -1.9 - 8.0 | 0.18 |
| **Amplitude 10 minutes (mm)** | 4.1 | -3.2 - 11.3 | 0.22 | 8.1 | -2.9 - 19.1 | 0.12 | 4.29 | -1.7 - 10.2 | 0.13 |
| **Maximum lysis (%)** | 0.7 | -3.8 - 5.1 | 0.73 | 0.1 | -5.7 - 5.9 | 0.96 | 0.16 | -0.5 - 0.8 | 0.35* |
| **EXTEM amplitude 5 minutes –**  **FIBTEM amplitude 5 minutes (mm)** | - | - | - | - | - | - | 0.1 | -0.2 – 0.4 | 0.38 |

* Log transformed data.

Abbreviations: CI = confidence interval. EXTEM = extrinsically activated test. FIBTEM = fibrin-based extrinsically activated test. INTEM = intrinsically activated test, IQR = interquartile range
